# Characterization of two-step deglycosylation via oxidation by glycoside oxidoreductase and defining their subfamily

**DOI:** 10.1038/srep10877

**Published:** 2015-06-09

**Authors:** Eun-Mi Kim, Joo-Hyun Seo, Kiheon Baek, Byung-Gee Kim

**Affiliations:** 1 School of Chemical and Biological Engineering, Institute of Molecular Biology and Genetics, and Bioengineering Institute, Seoul National University, Seoul, 151-742, Korea

## Abstract

Herein, we report a two-step deglycosylation mediated by the oxidation of glycoside which is different from traditional glycoside hydrolase (GH) mechanism. Previously, we reported a novel flavin adenine dinucleotide (FAD)-dependent glycoside oxidoreductase (FAD-GO) having deglycosylation activity. Various features of the reaction of FAD-GO such as including mechanism and catalytic residue and substrate specificity were studied. In addition, classification of novel FAD-GO subfamily was attempted. Deglycosylation of glycoside was performed spontaneously via oxidation of 3-OH of glycone moiety by FAD-GO mediated oxidation reaction. His493 residue was identified as a catalytic residue for the oxidation step. Interestingly, this enzyme has broad glycone and aglycon specificities. For the classification of FAD-GO enzyme subfamily, putative FAD-GOs were screened based on the FAD-GO from *Rhizobium* sp. GIN611 (gi 365822256) using BLAST search. The homologs of *R*. sp. GIN611 included the putative FAD-GOs from *Stenotrophomonas* strains, *Sphingobacterium* strains, *Agrobacterium tumefaciens* str. C58, *and etc*. All the cloned FAD-GOs from the three strains catalyzed the deglycosylation via enzymatic oxidation. Based on their substrate specificities, deglycosylation and oxidation activities to various ginsenosides, the FAD-GO subfamily members can be utilized as novel biocatalysts for the production of various aglycones.

Glycosides play numerous important roles in living organisms such as anti-oxidant, anti-cancer, anti-aging and anti-inflammation ingredients^1^,^2^. Many plants often produce and store various glycosides as biologically inactive form, but their deglycosylation improves their bioavailabilities and/or biological activities of glycosides^3^,^4^. Among various deglycosylation methods, enzymatic deglycosylation is the most preferred method, because it is substrate specific, produces less byproduct and gives high yields. Glycoside hydrolases are mostly often used for the hydrolysis of the glycosidic linkage of glycosides, following one of the two mechanisms, i.e. retaining or inverting mechanism^5^,^6^,^7^. In recent years, some interesting redox enzymes requiring NADH have been discovered to show deglycosylation activities, following different mechanisms^8^,^9^,^10^,^11^. These interesting enzymes were classified as glycoside hydrolase family 4 (GH4) and family 109 (GH109)^12^,^13^,^14^,^15^. Especially, GH4 enzymes hydrolyze the glycosidic linkage of 6-phospho-glycosides via redox-elimination, which involves a hydride abstraction at the C3 hydroxyl group of the substrate by enzyme-bound NAD^+^cofactor, thus oxidize the C3 hydroxyl group to a ketone. Such an oxidation acidifies the C2 proton, which is subsequently deprotonated by an enzymatic base^8^. Such enzymes require cofactor NAD^+^and divalent metal ion for their catalytic activity^16^,^17^,^18^,^19^, so that, their catalytic mechanisms are significantly different from those of general glycoside hydrolases. This unusual catalytic mechanism related to the oxidation of sugar moiety gave an inspiration to the identification and characterization of our FAD-dependent glycoside oxidoreductase (FAD-GO) reaction from *R*sp. GIN611^20^, which was reported from our previous research. The FAD-GO did not show the dependency of NAD^+^and divalent metal ion unlike the enzymes in family GH4. And the FAD-GO has no sequence similarity with GH4 family. Therefore, to characterize such an interesting reaction of FAD-GO, we have investigated the reaction of this enzyme more in detail, and carried out the identification of the subfamily of these FAD-GOs which would be a branch of glucose-methanol-choline oxidoreductase family.

## Material and Method

### Chemicals

Compound K (CK) (50%) was prepared by enzymatic biotransformation followed by the method of Kim *et al*.^21^. Ginsenosides Rb2, Rb3, Rc, Rd, F2, Rh2, *S*-protopanaxadiol (PPD(S)), Re and F1 were purchased from LKT Laboratories Inc. (St Paul, MN) and ginsenoside Rb1 was purchased from Wako Pure Chemical Co., Ltd. (Osaka, Japan). Glycyrrhizic acid was purchased from Sigma-Aldrich. Camelliaside and icariin was obtained from Amorepacific Corporation. All solvents for high-performance liquid chromatography (HPLC) analysis were used HPLC grade from Duksan Pure Chemical (Ansan, Republic of Korea). Ethanol and ethyl acetate was purchased from Merck-chemical (Darmstadt, Germany). 2, 5-dihydroxybenzoic acid (DHB), *p*-nitrophenyl-β-D-glycopyranoside and all chemicals were purchased from Sigma.

### Site-directed mutagenesis

Predicted structure of FAD-GO was constructed using SCONSIS^22^. Percent similarities between FAD-GO and each template sequence were calculated on the assumption that the aligning of similar amino acids is matched. Groups of similar amino acid are as follows: ‘aa1′:(G, A, V, L, I), ‘aa2′:(F, Y, W), ‘aa3′:(C, M), ‘aa4′:(S, T), ‘aa5′:(K, R, H), ‘aa6′:(D, E, N, Q), ‘aa7′:(P). 2jbvA (choline oxidase from *Arthrobacter globiformis*, best template, 28% similarity), 1cf3A (glucose oxidase from *Aspergillus niger*, 25% similarity), 1gpeA (glucose oxidase from *Penicillium amagasakiense*, 26% similarity), 1qjnA (aryl-alcohol oxidase from *Pleurotus eryngii*, 27% similarity) and 1ju2A (hydroxynitrile lyase from *Punus dulcis*, 25% similarity) were used as templates. FAD from the best template (A chain of 2JBV (2jbvA), choline oxidase from *Arthrobacter globiformis*) was incorporated to the active site using the MODELLER hetero-atom option. Key residues were predicted by the superposition of the model of FAD-GO onto 2jbvA. Because His351 and His466 are active site bases in FAD dependent choline oxidase (2jbvA)^23^, the corresponding residues in the FAD-GO were searched by structural alignment. Structure alignment was performed using PyMOL (http://www.pymol.org). Energy-minimized 3D structure of CK was constructed using Chem3D ultra 8.0 (Cambridgesoft, MA, USA). 100 docking poses were generated using Autodock 3.0^24^.

Following nucleotide sequences were used for the site-directed mutagenesis. H493F; 5′- CGC ACC CGG TAT GGG TAT TTT CGA AAT GGG AAC GGC GCG C-3′(forward direction) and 5′-GTT CCC ATT TCG AAA ATA CCC ATA CCG GGT GCG TAG CTG C-3′ (reverse direction). H493A; 5′- CGC ACC CGG TAT GGG TAT TGC CGA AAT GGG AAC GGC GCG C-3′ (forward direction), 5′- GTT CCC ATT TCG GCA ATA CCC ATA CCG GGT GCG TAG CTG C-3′ (reverse direction). These primers were used to replace His residue at positions 493 with Phe or Ala. Mutants were introduced by a PCR-based technique using *pfu* DNA polymerase. The PCR products were treated with *DpnI* and propagated into *Escherichia coli* DH5α cells. The obtained plasmids were sequenced to confirm the mutation on DNA sequences of ORF. Mutated recombinant plasmids were transformed into *E. coli* Rosetta gami2 (DE3, pLysS) for expression of mutant proteins. The activities of two mutants (H493F, H493A) were analyzed by HPLC.

### GC-MS analysis of oxidized glucose

After the reaction termination, the reaction mixture was extracted with ethyl acetate to acquire oxidized glucose, and the aqueous phase containing the oxidized glucose was collected. The collected sample was dried by vacuum evaporator. The prepared oxidized glucose was dissolved with trimethylsilane (TMS) solution for 10 min at room temperature for its derivatization. TMS derivative was analyzed by GC-MS (ITQ1100TM-GC/MS^n^, Thermo Scientific, USA) using a non-polar capillary column (5% phenyl methyl siloxane capillary 30 m × 250 μm i.d., 0.25 μm film thickness) and a linear temperature gradient (60 °C 1 min, temperature gradient of 30 °C/min to 250 °C, hold for 10 min, then 1 °C/min to 275 °C, and hold for 3 min). The injector port temperature was 100 °C. Mass spectra were obtained by electron impact ionization at 70 eV.

### Characterization of FAD-GOs

To analyze the profile of oxidized intermediates and deglycosylated products by oxidoreductases, matrix assisted laser desorption/ionization-time of flight (MALDI-TOF) mass spectrometry (Bruker Datonics Biflex IV, Bremen, Germany) was used. The reaction mixture was incubated at 37 °C for 3 h and 12 h with 50 mM sodium phosphate buffer (pH 7.0). The reaction mixture contained 100 μM substrate and 300 μl (1.5 mg/ml) purified enzyme in 1 ml reaction volume. After reaction, reactants were extracted with 1 ml ethyl acetate. The extracted samples went through evaporation using a vacuum concentrator (Biotron, Seoul, Republic of Korea). The dried reaction samples were dissolved in ethanol, and then analyzed by MALDI-TOF using 2, 5-dihydroxybenzoic acid as a matrix. The extracted samples were analyzed using MALDI-TOF mass spectrometry.

To analyze spontaneous reaction condition, oxidized CK was prepared using fraction collector during HPLC running of the reaction mixture. The fractionated oxidized CK was deglycosylated in 1 mM NaOH solution, pH 8.0, pH 7.0 and pH 6.0 sodium phosphate buffers and ethanol. After incubation for 3 h, the reaction mixtures were analyzed using HPLC.

### Screening and sequence anaylsis of putative FAD-GOs

To select the putative glycoside oxidoreductase, BLAST search against NR database was perfomed using FAD-GO from *R*. sp. GIN611 as a seed sequence. Six putative highly homologous oxidoreductases were selected from three species (one *Agrobacterium tumefaciens*, three *Stenotrophomonas sp*. and two *Sphingobacterium sp*.). Multiple alignments of three putative oxidoreductases from *Stenotrophomonas* species and two putative oxidoreductases from *Sphingobacterium* sp. were performed respectively using ClustalX. Using multiple alignment result, forward and backward primers were designed from conserved N-termincal and C-terminal sequence for the cloning of oxidoreductase from *Stenotrophomonas maltophilia* GIN612 and *Sphingobacterium multivorum* GIN723 (Table 1). Primers were used to amplify the coding gene of putative oxidoreductase form genomic DNA of strains. To identify the correct nucleotide sequence of 5′ and 3′ end, inverse PCR was performed.

Homologous sequences of FAD-GO from R. sp GIN611 were collected from RefSeq database using BLASTP. Total 49 sequences were retrieved. We added four sequences screened in this study to collected homologous sequence set. After the multiple sequence alignment of 53 sequences using ClustalX with default gap penalties, phylogenetic tree was constructed using TreeView.

### Cloning and expression of three glycoside oxidoreductases

The pETDuet-1-S was constructed with small subunit from *R*. sp. GIN611^20^. The pETDuet-1-S vector was used for the construction of the plasmids varying oxidoreductase genes (*Agrobacterium tumefaciens* str. C58, *Stenotrophomonas maltophilia* GIN612 and *Sphingobacterium multivorum* GIN723). Primers to clone three genes are listed in Table S1. All the primers were designed for protein to have His_6_-tag at the N-terminal. *S. maltophilia* GIN612 and *S. multivorum* GIN723 was screened in this study and *A. tumefaciens* str. C58 were purchased from ATCC (Manassas, VA, USA). The genomic DNA was prepared using G-spin and it was used as the template for the amplification of putative oxidoreductase genes. The coding region of oxidoreductases was amplified by PCR and the PCR products were digested with *BamHI* and *HindIII*. The digested PCR products were cloned into the multiple cloning sites of pETDuet-1-S. The pETDuet-1-S construct of the oxidoreductase was introduced into *E. coli* Rosetta-gami 2 (DE3, pLysS). Recombinant proteins were overexpressed in Luria-Bertani (LB) media containing 100 μg/ml of ampicilin at 20 °C for 12 h. 0.5 mM IPTG was added at OD_600_ 0.4 ~ 0.6. The cells were harvested by centrifugation at 7,000 g for 15 min at 4 °C. The cell pellets were washed with Phosphate buffered saline, and were resuspended in 5 ml, 50 mM Tris-HCl buffer (pH 7.4) containing 1 mM phenylmethanesulfonylfluoride (PMSF) and 1 mM dithiothreitol (DTT). The crude extract obtained by ultrasonic disruption and cell debris was removed by centrifugation at 14,000 g for 30 min at 4 °C. Expressed oxidoreductases were purified from the crude extract by Ni-NTA affinity purification method (QIAGEN Korea, Seoul, Republic of Korea)

## Result and Discussion

### His493 is catalytic base to oxidize glycone moiety

**Figure 1:**
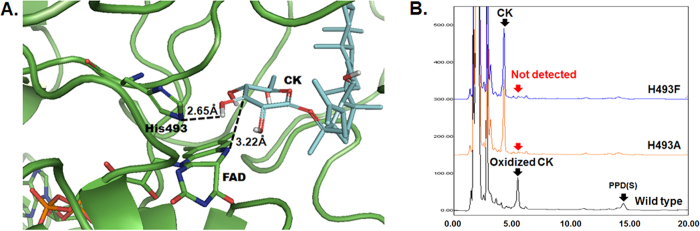
A The predicted active site structure of FAD-GO by homology modeling and protein-ligand docking simulation. Glucose in CK is placed between His493 and incorporated FAD. In protein, green-colored, blue-colored, red-colored and orange-colored atoms are carbon, nitrogen, oxygen and phosphorus, respectively. In CK, cyan-colored and red-colored atoms are carbon and oxygen, respectively. **B** HPLC chromatogram of enzyme reactions using wild type, H493F mutant or H493A mutant enzyme. Mutant strains did not produce oxidized CK and PPD(S). His493 is a key catalytic residue to oxidize glycone moiety in glycoside.

According to our previous research, FAD-GO from *R*. sp. GIN611 catalyzed deglycosylation of the ginsenoside via oxidation, and the mass spectra of a reaction intermediate from every substrate showed a peak of 2 Da lower than the expected mass (m/z) of the substrate, strongly suggesting the removal of two hydrogen atoms. To identify the possible basic residues abstracting a proton from the C3 hydroxyl groups of the substrate, structure of FAD-GO was predicted. Quaye *et al*.^23^ reported that His351 and His466 of 2jbvA are the active site bases. In addition, recent study reported that the proton abstraction by active site base was the first step of the reaction^25^,^26^,^27^. Therefore, the corresponding residues of His351 and His466 of 2jbvA were strongly expected as catalytic base and searched in the active site of FAD-GO. His493 of FAD-GO was structurally aligned to the His466 of 2jbvA. The structure alignment of the predicted structures of FAD-GO and 2jbvA showed that there were no basic residues at or near the corresponding position of His351 of 2jbvA. Therefore, His493 was predicted the active site base carrying out the proton abstraction in the first step of reaction. In Fig. 1A showing the protein-ligand docking result, His493 is located near the FAD and 2.65 Å away from the hydrogen atom of 3-hydroxyl group of the glucose of CK. Therefore, His493 was expected to be the catalytic residue responsible for the deprotonation reaction. To verify the importance of His493, site-directed mutagenesis was performed to make H493F and H493A mutant. H493F and H493A mutations were designed to see the effect of the absence of hydrogen bonding caused by the aromatic side chain of phenylalanine, and the effect of the deletion of the histidine residue on the enzyme activity, respectively. Both mutants completely lost their activities of deglycosylation (Fig. 1B). Moreover, the reactions using the two mutants did not produce the oxidized CK. These results indicate that the glucose oxidation by His493 of FAD-GO would be an initiating step of the overall deglycosylation.

### Characterization of two-step deglycosylation

**Figure 2:**
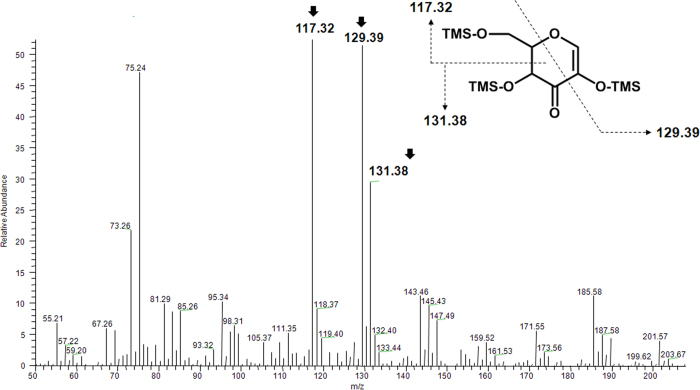
A fragmentation pattern of the C3 hydroxyl group oxidized glucose generated as a reaction intermediate by FAD-GO from *R*. sp. GIN611 by GC-MS/MS. Major three peaks were matched with fragment ion of oxidized glucose at 3-OH group.

**Figure 3:**
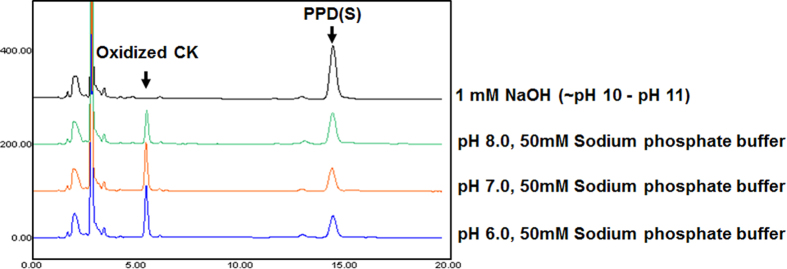
Deglycosylation of oxidized CK in different pH condition. By increasing pH, deglycosylation was performed faster and at the most basic condition (1 mM NaOH solution, pH 10-11), the oxidized CK was deglycosylated to PPD(S) completely.

**Figure 4:**
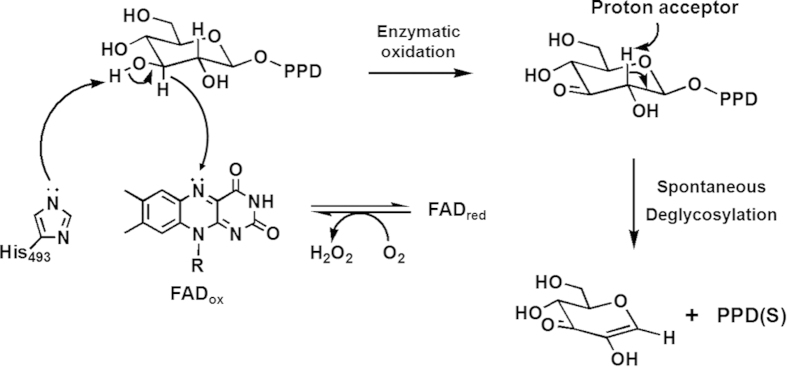
Proposed two-step deglycosylation by glycoside oxidoreductase. Two-step deglycosylation of FAD-dependent glycoside oxidoreductase is revealed that hydrolysis of glycosidic bond occur spontaneously via enzymatic oxidation at 3-OH. And His493 is catalytic residue to abstract the hydrogen of 3-OH.

To verify the oxidation position at glucose, the oxidized glucose released after spontaneous deglycosylation reaction was analyzed by GC-MS/MS. In Fig. 2, the spectrum shows the fragmentation pattern of the oxidized glucose. In the mass spectrum, three peaks at m/z 117.32, 129.39 and m/z 131.38 were detected as major peaks. Compared with the estimated fragmentation pattern, major three peaks of the GC-MS/MS spectrum matched with them. If the oxidation reaction occurs at other hydroxyl groups of glucose, these mass values would not be detected. These GC-MS/MS results support the deprotonation of C3 hydroxyl group and the formation of ketone at C3 position. Theoretically, if the C3 hydroxyl group is converted to ketone, the C2 hydrogen becomes acidic. To cleave the glycosidic bond, the C2 hydrogen has to be removed as a proton form. The removal of C2 hydrogen can be occurred by basic residue. However, we could not find any basic residues around the C2 hydrogen in the active site of predicted structure. From this analysis, we assumed that the abstraction of C2 hydrogen may be irrelevant to the enzymatic action. We examined the necessity of the proton acceptor by using the reaction intermediate (i.e. C3-keto form of glucose) fractionated by HPLC and subsequently treated using buffers without enzyme. When the collected reaction intermediate was treated with 1 mM NaOH or 50 mM sodium phosphate buffer solution with different pH, the reaction intermediate was converted into PPD(S), and the oxidized CK containing oxidized glucose moiety was generated at the same time in all the cases. Interestingly, if the pH is higher, the deglycosylation reaction rate was increased (Fig. 3). At the most basic condition (1 mM NaOH solution, pH 10 - 11), the oxidized CK was deglycosylated to PPD(S) completely. This result means that C2 proton abstraction occurred non-enzymatically. The spontaneous elimination of PPD(S) was expected due to the removal of the acidic C2 proton of the oxidized CK induced by the formation of the ketone group at C3 position. In other words, PPD(S) can be cleaved off by the formation of more stabilized form of the oxidized glucose due to the formation of a conjugated double bond in it in basic condition. Therefore, when CK is used as a starting substrate, the oxidized glucose moiety is removed from CK and then PPD(S) is produced. And, in our previous work, it was proved that FAD-GO is a FAD dependent enzyme, which is homologous to glucose-methanol-choline oxidoreductase (GMC oxidoreductase)^20^. According to the FAD-dependent enzyme mechanism, oxidized FAD (FAD_ox_) is generally regenerated from reduced FAD (FAD_red_) through the hydrogen transfer to dissolved oxygen (O_2_) generating a hydrogen peroxide (H_2_O_2_)^23^,^28^,^29^. Taken all together, we characterized two-step deglycosylation mechanism of FAD-GO like Fig. 4.

### Broad substrates spectrum of FAD-GO from *Rhizobium* sp. GIN611

**Figure 5:**
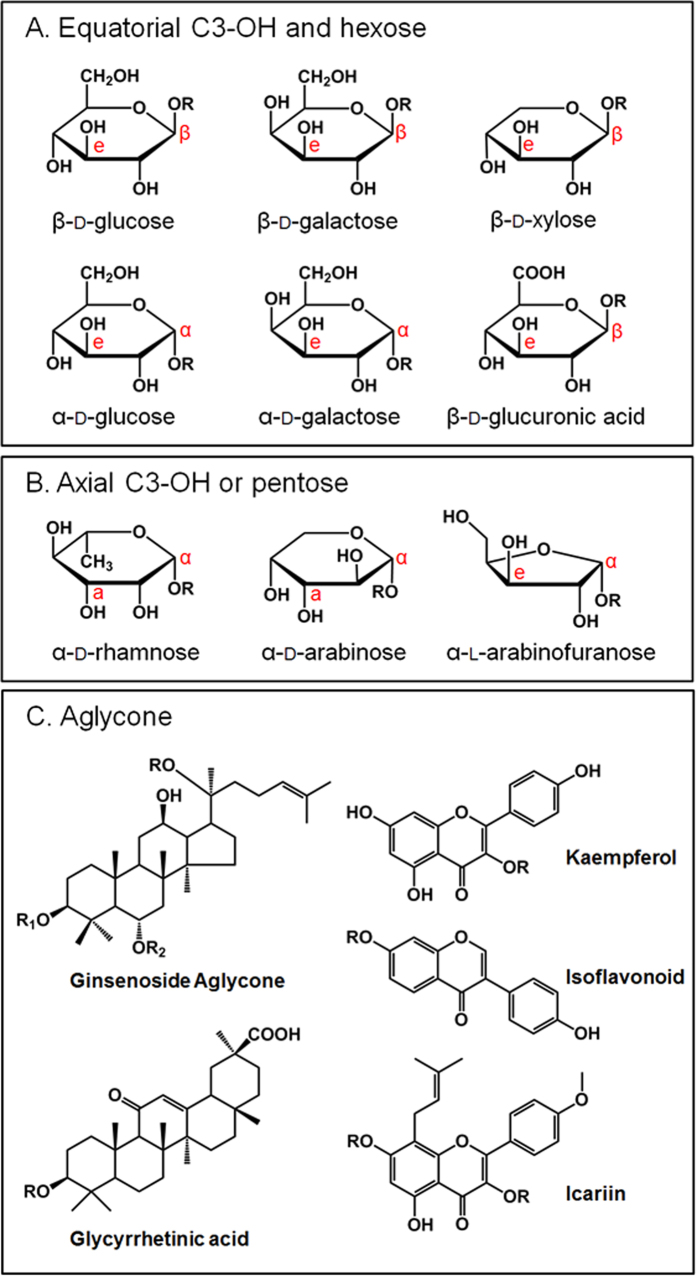
Broad spectrum of substrates. **A** active glycone. **B** non-active glycone **C** active aglycone. Ginsenoside aglycone (PPD group: R1: glycone, R2:H, PPT group: R1:H, R2: glycone) R: Glycone, e: equatorial, a: axial, α: alpha linkage, β: beta linkage

In our previous research, to determine the substrate specificities of FAD-GO, various substrates were examined^20^. Here, additional substrate (glycyrrhizin), which has glucuronic acid as glycone moiety was used. Its substrate specificities of glycone and aglycone were presented in Fig. 5. Glycosides are generally divided into two parts, i.e. aglycone and glycone parts. The glycone is consisted of single or several sugar moieties. In general, glycoside hydrolase shows a narrow but high specificity on the glycosyl moiety, aglycone structure, and glycosidic bond, respectively. On the contrary, our FAD-GO from *R*. sp. GIN611 had broad substrate spectrum on glycone, aglycone and glycosidic linkage. To explain deglycosylation mechanism by oxidation, we focused on the conformation of C3 hydroxyl group of sugar moiety of substrate. FAD-GO showed its activity on the substrates whose C3 hydroxyl group was in equatorial position. However, FAD-GO has no specificity on the types of glycosidic bond (Fig. 5A and Table S2). And also, FAD-GO didn’t show any activities on the glycosides with axial conformation of C3 hydroxyl group or five-membered ring sugar moiety either even though C3 hydroxyl group is equatorial (Fig. 5B). From substrate docking analysis, it is likely that the axial C3 hydroxyl group cannot participate in the oxidation reaction because the hydrogen atom of axial C3 hydroxyl group is too distant from the H493 to be abstracted (data not shown). Therefore, for the oxidation reaction by FAD-GO, an equatorial hydroxyl group at C3 position and pyranose ring formation in sugar moiety are necessary conditions. Figure 5C showed some examples of the active substrates having various aglycone types. FAD-GO had some activities on all of these substrates, indicating that FAD-GO has broad specificity on the types of aglycone. From these results, the active site of FAD-GO is highly focused and optimized for the recognition of six-membered ring sugar moiety having an equatorial C3 hydroxyl group rather than the recognition of other parts of substrates (i.e. aglycone and glycosidic linkage).

### Screening and sequence anaylsis of putative FAD-GOs

We were curious on the real function of this enzyme and their homologs, since this enzyme are very similar to glucose-methanol-choline (GMC) oxidoreductase which is a family of enzymes including choline dehydrogenase, methanol oxidase, and glucose oxidase. If the major function of this enzyme and its homologs are deglycosylation of glycosides, it should be newly defined as one of subfamilies of GMC oxidoreductase family as a glycoside oxidoreductase. To find the putative glycoside oxidoreductase, BLAST search against NR database was perfomed using FAD-GO from *R*. sp. GIN611 as a seed sequence. Six putative highly homologous oxidoreductases were selected. Those are oxidoreductase (gi|15890606, identity: 93%) from *Agrobacterium tumefaciens* str. C58, putative oxidoreductase (gi|190573214, identity: 75%) from *Stenotrophomonas maltophilia* K279a, oxidoreductase (gi|254522562, identity: 75%) from *Stenotrophomonas* sp. SKA14, glucose-methanol-choline oxidoreductase (gi|194364820, identity: 75%) from *Stenotrophomonas maltophilia* R551-3, oxidoreductase (gi|241893181, identity: 67%) from *Sphingobacterium spiritivorum* ATCC 33861 and oxidoreductase (gi|227540026, identity: 67%) from *Sphingobacterium spiritivorum* ATCC 33300. Interestingly, *Stenotrophomonas* sp. GIN612 (not reported) and *Sphingobacterium* sp. GIN723 were screened for β−glucosidase activity for ginsenosides in our previous research^30^. Therefore, those two strains and the first hit strain *A. tumefaciens* str. C58 were selected for this study.

For the isolation of the putative FAD-GO from *S. maltophilia* GIN612, multiple alignments of the three putative oxidoreductases from *Stenotrophomonas* species were performed using ClustalX (data not shown). Three sequences are almost identical. Especially, N-terminal and C-terminal sequences are highly conserved. Therefore, forward and backward primers for the cloning of oxidoreductase from *S. maltophilia* GIN612 were designed from conserved terminal sequence of three oxidoreductase sequences (Table 1). Two primers were used for PCR of genomic DNA from *S. maltophilia* GIN612 to find the encoding gene sequence of putative oxidoreductase. PCR product of an estimated size (1.7 kb) was obtained. From the sequencing and BLAST results, PCR product was expected to be a putative glycoside oxidoreductase gene. The DNA and translated amino acid sequences were presented in Fig. S1. The putative oxidoreductase from *S. maltophilia* GIN612 showed average 98% sequence identity to the three sequences from *Stenotrophomonas* species. For the cloning of a homolog oxidoreductase from *S. multivorum* GIN723, known two oxidoreductases from *Sphingobacterium* species in BLAST result were compared. 5′ and 3′ nucleotide sequences of the two *Sphingobacterium* species in BLAST result were identical. Therefore, these sequences were used as forward and backward primers (Table 1). The two primers were used for PCR of genomic DNA from *S. multivorum* GIN723 to amplify the coding gene of putative oxidoreductase. PCR products were obtained and sequenced. To identify the correct nucleotide sequence of 5′ and 3′ end, inverse PCR was performed. The correct full sequence of putative oxidoreductase from *S. multivorum* GIN723 was obtained. Putative oxidoreductase from *S. multivorum* GIN723 showed average 86% sequence identity to the two oxidoreductases from *Sphingobacterium* species. Two amino acids in N-terminus are different from the amino acid sequence in the primers. The DNA and translated amino acid sequences were presented in Figure S2. In Figure S3, we could propose that the subfamily containing from oxidoreductase Asp_PR1_1 to oxidoreductase_Sspi_ATCC33861_2 is FAD-GO subfamily branched from GMC oxidoreductase.

### Nucleotide sequence accession numbers

The two enzymes from *S. maltophilia* GIN612 and *S. multivorum* GIN723 have been submitted to GenBank and given accession numbers are KJ922518 and KJ922519, respectively.

### Characterization of the three glycoside oxidoreductases

**Figure 6:**
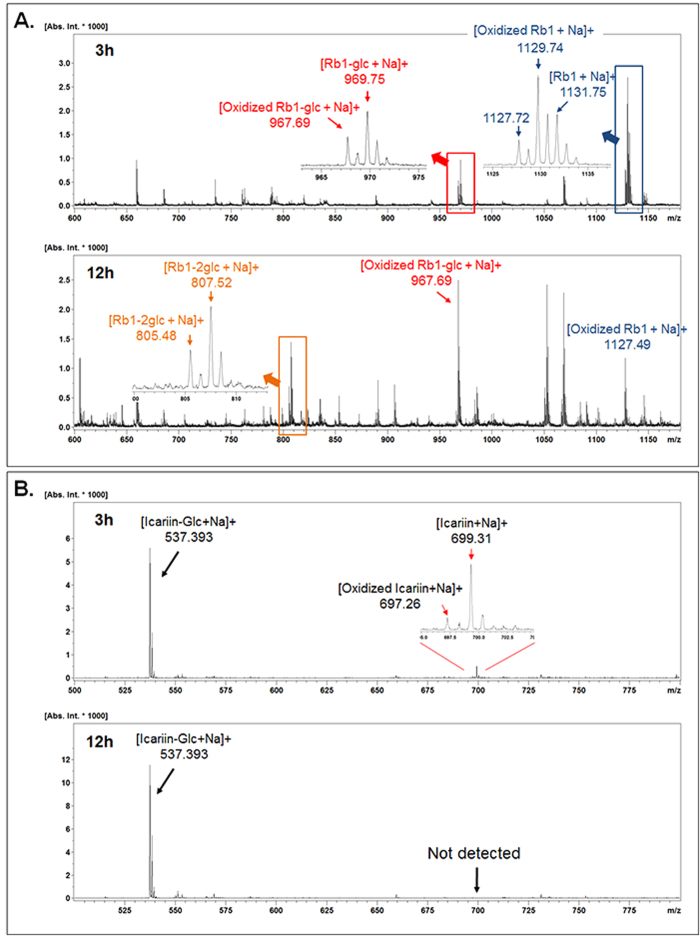
Reaction analysis by MALDI-TOF. **A** Ginsenoside Rb1 reaction by S. multivorum GIN723, **B** Icariin reaction by oxidoreductase from *S*. *maltopholias* GIN612.

Glycoside oxidoreductases from the three strains shown above were cloned to study the FAD-GO enzyme subfamily related to deglycosylation of glycoside. The substrate specificity of FAD-GO from *A. tumefaciens* str. C58 and *S. multivorum* GIN723 were the same as that of the FAD-GO from *R*. sp GIN611^20^. FAD-GO from *S. maltophilia* GIN612 did not show deglycosylation activity to ginsenoside CK and F2 that glucose moieties directly conjugated at C3 and C20 positions of ginsenoside. Therefore, it seems to have deglycosylation activity for the terminal glucose at the end of the multiple sugars moiety. Figure 6 shows mass spectra of ginsenoside Rb1 reaction by oxidoreductase from *S. multivorum* GIN723 and mass spectra of icariin reaction by oxidoreductase from *S. maltophilia* GIN612. It indicates that oxidoreductases always generated the mass spectra peaks of the molecular mass of substrates with 2 Da less, which are oxidized intermediates. At the same time, deglycosylated peak was detected. Additionally, some of the mass spectra of the reaction mixtures are presented in Figure S4. Intermediates and products profile for deglycosylation by three oxidoreductases are presented in Table 2. According to these results, three enzymes followed the same enzyme activity of FAD-GO oxidoreductase from *R*. sp GIN611

One interesting observation to remark is that the three putative oxidoreductases have a special gene arrangement with the presence of their partners in the downstream of oxidoreductases, the relatively partner proteins annotated as twin arginine translocation protein (TAT protein) appeared to have a chaperone-like function for their activities (Figure S5). In *A. tumefaciens* str. C58 and *S. maltophilia* GIN612, the ATG starting codon of chaperone-like protein was overlapped with the TAA stop codon of oxidoreductase. In the case of *S. multivorum* GIN723, a dehydrogenase gene is inserted between the genes of the two proteins, but the two proteins are located quite close to each other. TAT proteins are key factors to translocate the substrate proteins in prokaryotes and generally considered to have a role in protein secretion and virulence^31^. Besides these activities, there is a paper reporting the chaperone activity of TAT translocase^32^. Like the report of Chan *et al*.(2006), we also observed that the putative TAT protein tends to promote the solubility of FAD-GOs. When the putative TAT protein from *R*. sp. GIN611was co-expressed, the soluble expression of the other three oxidoreductases (*A. tumefaciens* str. C58 and *S. maltophilia* GIN611) were highly enhanced. These results suggest that putative TAT protein from *R*. sp. GIN611 may have a role of chaperone specifically for the FAD-GOs.

## Conclusion

Throughout this work, it was proved that FAD-assisted oxidation of sugar can lead to the deglycosylation of glycosides. The abstraction of the two hydrogen atoms of glucose (or other sugars) by a base (for example, His493 in FAD-GO) and FAD agrees well with the experimental analysis by mass spectrometry. Throughout sequence analysis, analysis of their enzyme activity and functional studies, we have tried to search homologous sequences, and identify a subfamily of FAD depedent glycoside oxidoreductase. Using the BLAST search, six putative oxidoreductases were selected from three genus (*Agrobacterium, Stenotrophomonas* and *Sphingobacterium*). Surprisingly, the microorganisms selected from both experimental screening and database search are pretty much the same. In the experimental screening, *S. maltophilia* GIN612 showed deglycosylation activity to ginsenosides including Rb1, and *S. multivorum* GIN723 showed the same activity for various ginsenosides including CK. Then, the similar glycoside oxidoreductases from the two strains were additionally identified in this study. The oxidoreductase sequence from *A. tumefaciens* str. C58 was already known, but its function as glycoside oxidoreductase was newly identified for the first time. All three putative oxidoreductases have glucose oxidase activity and subsequent, deglycosylation activity to ginsenosides, which made them to look like glucosidase.

In conclusion, FAD dependent glycoside oxidoreductase was characterized and additionally, three related enzymes and their substrate specificities were identified. In terms of glucosidase activity, the enzyme of FAD-GO subfamily has great advantages over other common hydrolases. Firstly, although it uses FAD cofactor, the cofactor regeneration is not a problem because FAD is automatically regenerated by molecular oxygen. Another advantage is that FAD-GO has broad substrate specificity only on the glycone type, but no specificity on glycone linkage and aglycone type, which makes FAD-GO more attractive in its industrial applications. Glycoside oxidoreductase appears to be a promising biocatalyst for the deglycosylation of glycoside in industrial processes such as food and pharmaceutical industry owing to its broad substrate specificity. This work has provided some sequence basis for the bioinformatic screening of additional FAD-GOs, and defined them as a FAD-GO subfamily of GMC oxidoreductase, which can be useful information for further study of the evolution of GMC oxidoreductase.

## Additional Information

**How to cite this article**: Kim, E.-M. *et al*. Characterization of two-step deglycosylation via oxidation by glycoside oxidoreductase and defining their subfamily. *Sci. Rep*. **5**, 10877; doi: 10.1038/srep10877 (2015).

## Supplementary Material

Supplementary Information

## Figures and Tables

**Table 1:** 5′ and 3′ nucleotide sequences of putative glycoside oxidoreductases and primers for analysis of coding gene.

Strains	5′-nucleotide sequence	3′-nucleotide sequence
*Stenotrophomonas maltophilia* K279a	ATGGCAGATAATCACTACGACGCC	GGACTGAAAGCGGGGAACCTCTGA
*Stenotrophomonas* sp. SKA14	ATGGCAGATAATCACTACGACGCC	GAACTGAAGGCGGGGAACCTCTGA
*Stenotrophomonas maltophilia* R551-3	ATGGCAGATAATCACTACGACGCC	GAACTGAAAGCGGGGAACCTCTGA
*Stenotrophomonas maltophilia* GIN612	**Forward ATGGCAGGTAATCACTACGACGCC**	**Backward GAACTGAAAGCGGGGAACCTCTGA**
*Sphingobacterium spiritivorum* ATCC 33861	ATGGCAGATAATGTATATGACGCA	GAGCTGAAAAAAGGAAACCTATAA
*Sphingobacterium spiritivorum* ATCC 33300	ATGGCAGATAATGTATATGACGCA	GAGCTGAAAAAAGGAAACCTATAA
*Sphingobacterium multivorum* GIN723	**Forward ATGGCAGATAATGTATATGACGCA**	**Backward GAGCTGAAAAAAGGAAACCTATAA**

**Table 2:** Intermediates and products list for deglycosylation by three oxidoreductases

Strains	A. tumefaciens str. C58		S. maltophilia GIN612		S. Multivorum GIN723	
Substrate	Intermediate	Final Product	Intermediate	Final Product	Intermediate	Final Product
**Rb1**	Oxidized Rb1	PPD(S)	Oxidized Rb1	F2	Oxidized Rb1	PPD(S)
**Rb2**	Oxidized Rb2	Compound Y	Oxidized Rb2	Deglycosylated Rb2	Oxidized Rb2	Compound Y
**Rb3**	Oxidized Rb3	PPD(S)	Oxidized Rb3	Gp-IX	Oxidized Rb3	PPD(S)
**Rc**	Oxidized Rc	Compound Mc	Oxidized Rc	Natoginsenoside Fe	Oxidized Rc	Compound Mc
**Rd**	Oxidized Rd	PPD(S)	Oxidized Rd	F2	Oxidized Rd	PPD(S)
**F2**	Oxidized F2	PPD(S)	N.D	N.D	Oxidized F2	PPD(S)
**CK**	Oxidized CK	PPD(S)	N.D	N.D	Oxidized CK	PPD(S)
**Rh2**	Oxidized Rh2	PPD(S)	N.D	N.D	Oxidized Rh2	PPD(S)
**Re**	Oxidized Re	Rg2	N.D	N.D	Oxidized Re	Rg2
**F1**	Oxidized F1	PPT(S)	N.D	N.D	Oxidized F1	PPT(S)
**Icariin**	N.D	N.D	Oxidized Icariin	Icariside	Oxidized Icariin	Icariside
**Camelliaside A, B**	N.D	N.D	Oxidized Camelliaside	6-O-Rha-Kampferol	N.D	N.D

^*^N.D: not detected, Substrate structure is presented Table S3.
