# Supplementary material for: Characterization of two-step deglycosylation via oxidation by glycoside oxidoreductase and defining their subfamily
Source: Sci Rep. 2015 Jun 9;5:10877. doi: 10.1038/srep10877 (PMC4650693; doi:10.1038/srep10877)
Supplement: Supplementary Information [file srep10877-s1.pdf]

Supporting Information

Characterization of two-step deglycosylation via oxidation by glycoside oxidoreductase and defining their subfamily

Eun-Mi Kim, Joo-Hyun Seo, Kiheon Baek and Byung-Gee Kim*

School of Chemical and Biological Engineering, Seoul National University, 151-742, Seoul, Korea

```
1 - ATGGCAGATAATCACTACGACGCCATTGTTGTTGGCTCGGGAATCAGTGGCGGTTGGGCG - 60
1 - M A D N H Y D A I V V G S G I S G G W A - 20

61 - GCGAAGGAGCTGACTGAAAAGGGCCTGAAGGTCCTGATGCTGGAACGTGGCCGTAACATC - 120
21 - A K E L T E K G L K V L M L E R G R N I - 40

121 - GAGCACGTCAAGGACTACGTCAACGCGATGAAGGAGGCGTGGGACTTCCCGCACCGCAAT - 180
41 - E H V K D Y V N A M K E A W D F P H R N - 60

181 - CGGCCAACGCAGGCGATGAAGGCCGACTTCCCGGTGCTGATGCGCGATTACGGCCTGGCC - 240
61 - R P T Q A M K A D F P V L M R D Y G L A - 80

241 - GAAAATCTGGAAGGAATGTGGGCCAACGAACAGGACTCGCCCTACATCGAGACCAAACGT - 300
81 - E N L E G M W A N E Q D S P Y I E T K R - 100

301 - TTCGACTGGTTCCGTGGCTACCACGTCGGCGGCCGCTCGCTGCTGTGGGGGCGGCAGAGT - 360
101 - F D W F R G Y H V G G R S L L W G R Q S - 120

361 - TATCGCTTCTCCGATCTGGATTTCGAGGCGAACCTCAAGGATGGGATCGCTGCTGACTGG - 420
121 - Y R F S D L D F E A N L K D G I A A D W - 140

421 - CCGATCCGCTACGCCGACATCGCGCCGTGGTACGACCATGTGGAAAGGTTCGCAGTCATC - 480
141 - P I R Y A D I A P W Y D H V E R F A V I - 160

481 - GCCGGCACGCGCGAAGGACTGGACGTGCTGCCGGACGGCGAATTCCTGCCGCCGATTCCC - 540
161 - A G T R E G L D V L P D G E F L P P I P - 180

541 - TTGAACATCGTCGAGAAGGATGTGGCCGCGCGTATCAAGAAGGCCTTTGGCGGAACGCGC - 600
181 - L N I V E K D V A A R I K K A F G G T R - 200

601 - CACATGATCCACTCGCGCACCGCCAACATCACCAAACCGATGCCCGAGCAGGGGCGCGTC - 660
201 - H M I H S R T A N I T K P M P E Q G R V - 220

661 - AACTGCCAGTACCGCAACAAGTGCATCCTGGGCTGTCCCTTCGGTGCCTACTTCTCGACC - 720
221 - N C Q Y R N K C I L G C P F G A Y F S T - 240

721 - CAGGCGGCGACGCTGCCAGCGGCGGTGAAGACCGGCAACCTGACCTTGCGTCCGTTCTCG - 780
241 - Q A A T L P A A V K T G N L T L R P F S - 260

781 - ATCGTCAAGGAAGTGCTCTATGACAAGGATCGCAAGCGTGCCCGCGGCGTGGAGGTCATC - 840
261 - I V K E V L Y D K D R K R A R G V E V I - 280

841 - GACGCCGAGACCGGGCAGACCTACCAGTACACGGCCAAGGTGATCTTCCTCAATGCGTCG - 900
281 - D A E T G Q T Y Q Y T A K V I F L N A S - 300

901 - TCGTTCAACTCGACCTGGCTGCTGATGAATTCGGCGACCGATGTATGGGACGGCGGGCTG - 960
301 - S F N S T W L L M N S A T D V W D G G L - 320
```

(Continued)

```
 961 - GGCTCTTCGTCCGGTGAGTTGGGGCACAACGTGATGGATCATCATTTCGGTGCGGGTGCC - 1020
 321 - G  S  S  S  G  E  L  G  H  N  V  M  D  H  H  F  G  A  G  A   - 340

1021 - TCGGGCCGGGTCGAGGGCTACGAAGACAAGTACTACTTCGGCCGCCGTCCCTGCGGTTTC - 1080
 341 - S  G  R  V  E  G  Y  E  D  K  Y  Y  F  G  R  R  P  C  G  F   - 360

1081 - TACATTCCGCGCTTCCGCAACGTCGCAGCCGACAAGCGTGGCTACCTGCGCGGGTTCGGC - 1140
 361 - Y  I  P  R  F  R  N  V  A  A  D  K  R  G  Y  L  R  G  F  G   - 380

1141 - TACCAGGGCGGTGCCAGCCGAAACGGCTGGTCGCGCGAGATCGCCGAGCTGAACATCGGC - 1200
 381 - Y  Q  G  G  A  S  R  N  G  W  S  R  E  I  A  E  L  N  I  G   - 400

1201 - GCCGACCTGAAGGAAGCGCTGACCGTGCCGGGTGACTGGCGCATCGGCATGACCGGGTTC - 1260
 401 - A  D  L  K  E  A  L  T  V  P  G  D  W  R  I  G  M  T  G  F   - 420

1261 - GGTGAAATGCTGCCGCACCACGACTATACAATCCGCCTGGACCACGACCGCAAGGACAAG - 1320
 421 - G  E  M  L  P  H  H  D  Y  T  I  R  L  D  H  D  R  K  D  K   - 440

1321 - TGGGGGCTGCCGGTGCTGGCGATGGACGTTGCCATGCGTGCCAACGAACTGGCGATGCGC - 1380
 441 - W  G  L  P  V  L  A  M  D  V  A  M  R  A  N  E  L  A  M  R   - 460

1381 - AAGGACATGGCCGCCGATGCTGCCGAAATGCTGGAGGCGGCCGGCGTCAAGGACGTGAAG - 1440
 461 - K  D  M  A  A  D  A  A  E  M  L  E  A  A  G  V  K  D  V  K   - 480

1441 - ATGCACGACAACGACTATGCGCCGGGCAAGGGCATCCACGAGATGGGGACCGCGCGCATG - 1500
 481 - M  H  D  N  D  Y  A  P  G  K  G  I  H  E  M  G  T  A  R  M   - 500

1501 - GGACGTGATCGCAAGAGCTCGGTACTGAACCAGCACAACCAGGTCTGGGATGCTCCCAAC - 1560
 501 - G  R  D  R  K  S  S  V  L  N  Q  H  N  Q  V  W  D  A  P  N   - 520

1561 - GTCTATGTGACCGACGGTGCCTGCATGACCTCCAGCGCCTGCGTGAATCCCTCGCTGACC - 1620
 521 - V  Y  V  T  D  G  A  C  M  T  S  S  A  C  V  N  P  S  L  T   - 540

1621 - TACATGGCGCTGACCGCGCGCGCGGCCGACCACGCGGTACGCGAACTGAAGGCGGGGAAC - 1680
 541 - Y  M  A  L  T  A  R  A  A  D  H  A  V  R  E  L  K  A  G  N   - 560

1681 - CTCTGA                                                       - 1686
 561 - L  *                                                         - 580
```

Figure S1. DNA and translated amino acid sequence of glycoside oxidoreductase from *S. maltophilia* GIN612.

1 - ATGGCAACAAATACTTATGACGCAATTGTAATCGGTTCGGGGATAAGTGGTGGATGGGCT - 60
1 - M A T N T Y D A I V I G S G I S G G W A - 20

61 - GCGAAAGAATTGACAGAGAAAGGGCTGAAAACAATTATGCTGGAGCGTGGTCGTAACATC - 120
21 - A K E L T E K G L K T I M L E R G R N I - 40

121 - GAACACATCAAAGATTATACTGCGCCAAATAAAAATCCATGGGAATGGCCACATGCTGGC - 180
41 - E H I K D Y T A P N K N P W E W P H A G - 60

181 - GGTCGTACGCAAAAAATGATCGAAGAGTATCCGGTTTTGCGTAGAGATTACCCATTGAAC - 240
61 - G R T Q K M I E E Y P V L R R D Y P L N - 80

241 - GAAAAAAACCTGGATTTTTGGGTAAATGAAAAGGAAAGCCCTTATACCGAAGTCAAACGT - 300
81 - E K N L D F W V N E K E S P Y T E V K R - 100

301 - TTTGATTGGTACCGTGGGTATCATGTTGGCGGTAGATCGCTGATGTGGGGAAGACAATCT - 360
101 - F D W Y R G Y H V G G R S L M W G R Q S - 120

361 - TATCGTTTAGGCGATTTGGATTTCGAAGCGAACTTAAAAGACGGTCACGGTGTAGACTGG - 420
121 - Y R L G D L D F E A N L K D G H G V D W - 140

421 - CCAATTCGTTATAATGAAATTGCCCCTTGGTATAGTTATGCGGAGAAATTTGCTGGTATC - 480
141 - P I R Y N E I A P W Y S Y A E K F A G I - 160

481 - TCCGGTAATCGTGACGGTGTACCATCTTTGCCAGATGGAGACTACATGCCTGCAATGGCG - 540
161 - S G N R D G V P S L P D G D Y M P A M A - 180

541 - ATGAATATTGTTGAAAAAGACTTAGCGGAGCGTTTGAAAAAACAATACGGCGGTCAACGT - 600
181 - M N I V E K D L A E R L K K Q Y G G Q R - 200

601 - CATTTCATCATGGGTAGAACAGCAAACATTACTGTTCCGCATCACGACCGTGTAAACTGT - 660
201 - H F I M G R T A N I T V P H H D R V N C - 220

661 - CAATATCAAAATCAATGTTGGTTGGGTTGTAACTTTGGTGCCTACTTCAGTACACAGTCG - 720
221 - Q Y Q N Q C W L G C N F G A Y F S T Q S - 240

721 - GCGACTCTTCCTGCAGCGAAAAAAACAAATAACTTGACATTACGCCCTTTTTCCATCGTA - 780
241 - A T L P A A K K T N N L T L R P F S I V - 260

781 - ACTAAAATTATCTACGATAAAAACACGAAAAAGGCAAAAGGTGTAGAAATTGTCGACGCT - 840
261 - T K I I Y D K N T K K A K G V E I V D A - 280

841 - GAAACAAATCAAACCTATGAATTTTTCGCAAAGGTCATCTTTGTTTGTGCATCTGCATTG - 900
281 - E T N Q T Y E F F A K V I F V C A S A L - 300

901 - AACTCAACGTGGGTATTGATGAATTCTGCCACAGATGTTTGGGAAGGCGGTCTGGGTAGC - 960
301 - N S T W V L M N S A T D V W E G G L G S - 320

(Continued)

```
961 - AGCAGTGGCGAATTGGGGCATAACCTGATGGATCACCATTTCCGTTGTGGTGCCGGAGGT - 1020
321 - S  S  G  E  L  G  H  N  L  M  D  H  H  F  R  C  G  A  G  G  - 340

1021 - AAGATTGATGGTTATTTGGATAGCTATGTGTATGGCCGCAGACCGACAGGTTTGTATGTA - 1080
341 - K  I  D  G  Y  L  D  S  Y  V  Y  G  R  R  P  T  G  L  Y  V  - 360

1081 - CCTCGTTTTGTAAACGTAGAAGGTGATACCAAAAAACGTGATTATGTTCGTGGATTTGGA - 1140
361 - P  R  F  V  N  V  E  G  D  T  K  K  R  D  Y  V  R  G  F  G  - 380

1141 - TACCAAGGTGCGGCAGGTCGCGGACGTTGGTCTGGTGCTGTTGCCGAGATGGAAGTGGGG - 1200
381 - Y  Q  G  A  A  G  R  G  R  W  S  G  A  V  A  E  M  E  V  G  - 400

1201 - GGTGCATGGAAAGATGCCATCTGTGAGCCAGGTGATTGGACCGTAGGTTTTACCGCTTTC - 1260
401 - G  A  W  K  D  A  I  C  E  P  G  D  W  T  V  G  F  T  A  F  - 420

1261 - GGAGAAACGTTACCTTATCACGAAAATAAAGTTACGCTTGATAAAAGCAAAAAAGATAAA - 1320
421 - G  E  T  L  P  Y  H  E  N  K  V  T  L  D  K  S  K  K  D  K  - 440

1321 - TGGGGATTACCTGTATTGTCATTTGACGCGGAGATCAAGGATAATGAATTGAAGATGCGT - 1380
441 - W  G  L  P  V  L  S  F  D  A  E  I  K  D  N  E  L  K  M  R  - 460

1381 - GGTGACATGCAGAATGAGATGAAAGAAATGTTGGAAAAAGTTGGCGTGAAAGATATCTAT - 1440
461 - G  D  M  Q  N  E  M  K  E  M  L  E  K  V  G  V  K  D  I  Y  - 480

1441 - ACCTACGATAATGTATATGGCTTTGGTCAAGGTATCCACGAGATGGGAACTGCCCGTATG - 1500
481 - T  Y  D  N  V  Y  G  F  G  Q  G  I  H  E  M  G  T  A  R  M  - 500

1501 - GGACGTGATCCAAAAACTTCCGTCTTAAATGGTAACAACCAAGTGTGGGATGCATTGAAC - 1560
501 - G  R  D  P  K  T  S  V  L  N  G  N  N  Q  V  W  D  A  L  N  - 520

1561 - GTCTTTGTGACTGATGGTGCTTGTATGACTTCTGCCGGTTGTGTCAACCCTTCATTAACG - 1620
521 - V  F  V  T  D  G  A  C  M  T  S  A  G  C  V  N  P  S  L  T  - 540

1621 - TACATGGCGCTTACTGCTCGTGCAGTTGACTTTGCAGTAAGTGAATTGAAAAAAGGTAAC - 1680
541 - Y  M  A  L  T  A  R  A  V  D  F  A  V  S  E  L  K  K  G  N  - 560

1681 - ATCTAA                                                       - 1686
561 - I  *                                                         - 580
```

Figure S2. DNA and translated amino acid sequence of glycoside oxidoreductase from *S. multivorum* GIN723.

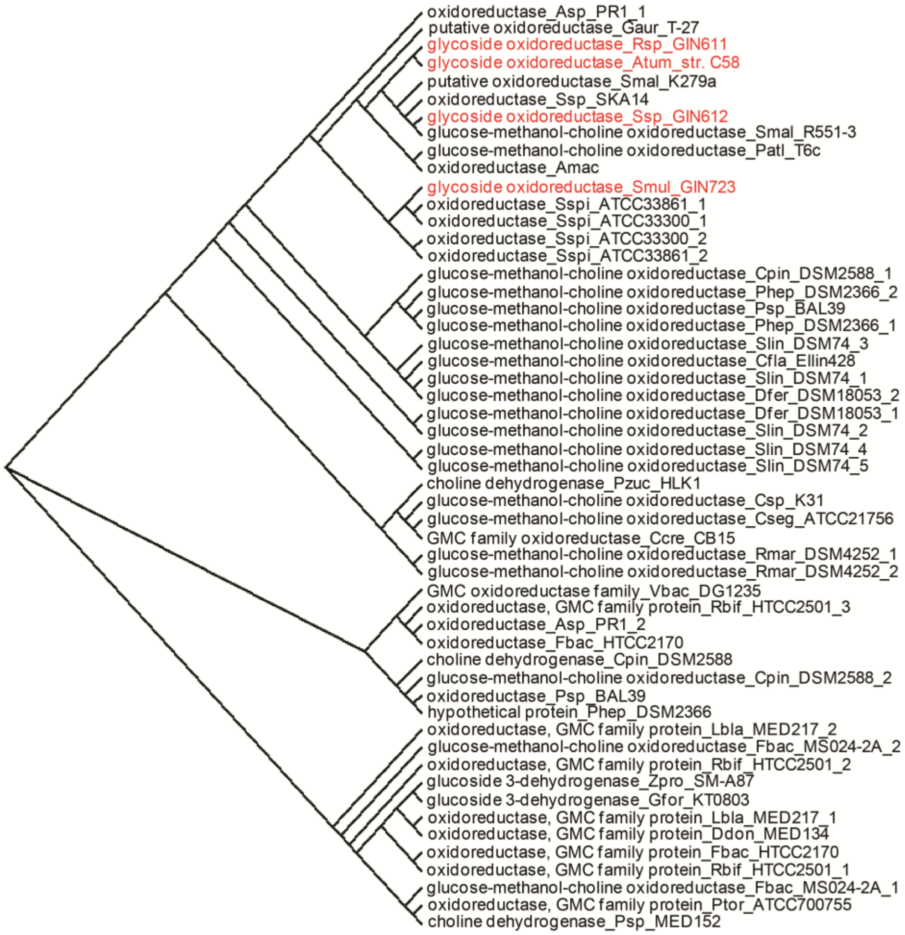

Figure S3. Phylogenetic tree analysis of glycoside oxidoreductases with neighbor sequences

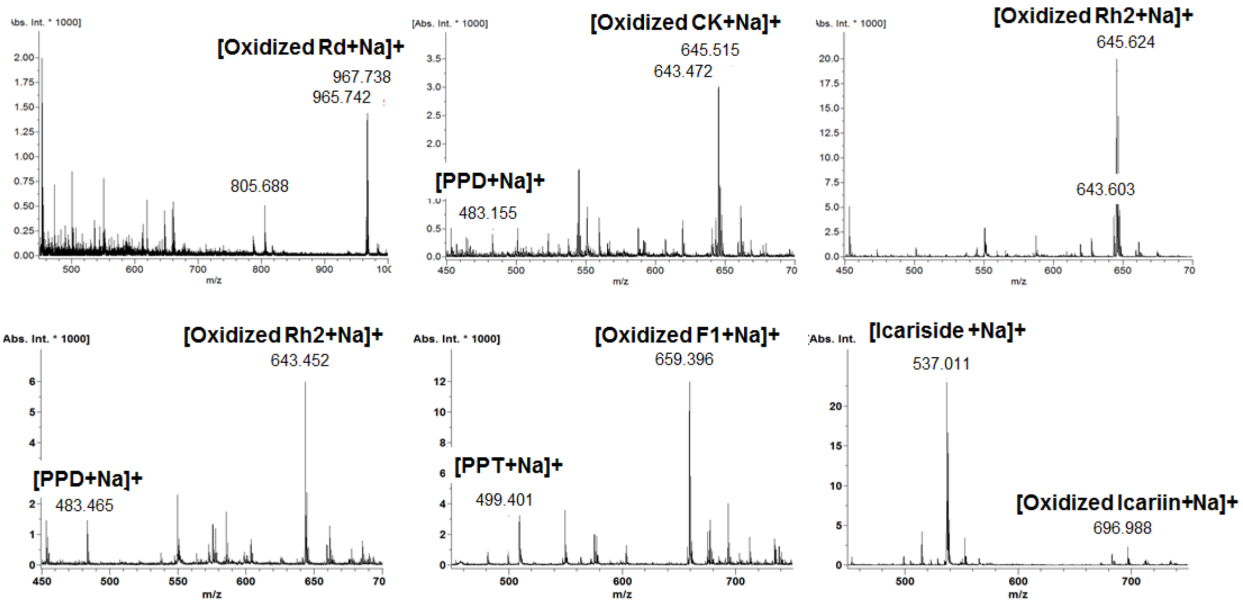

Figure S4. Mass spectra of reaction mixture

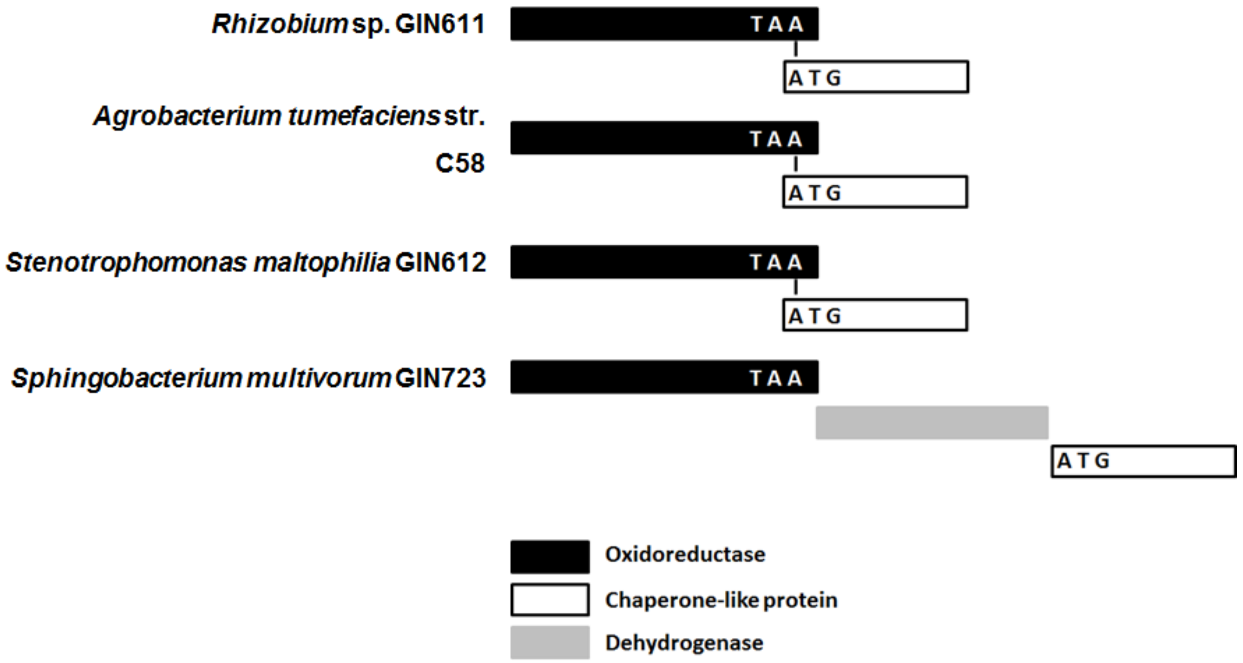

Figure S5. Gene arrangement of three FAD-GOs. A. Gene structure of *A. tumefaciens* str. C58. B. Gene structure of *S. maltophilias* GIN611 C. *S. multivorum* GIN723

SUPPLIMENTARY TABLE

Table S1. Primers for cloning

Strains		Primer for cloning (underline indicates the restriction enzyme site)
Agrobacterium tumefaciens str. C58	Forward	5′- ATATATGGATCCGATGGCAGACAATCATTATGATGCGA-3′
	Backward	5′- ATATATAAGCTTTCACAGGTTCCCCTTCTTACGTTCG-3′
Stenotrophomonas maltophilia GIN612	Forward	5′-ATATATGGATCCGATGGCAGGTAATCACTACGACGCCA-3′
	Backward	5′- ATATATAAGCTTTCAGAGGTTCCCCGCTTTCAGTTCG-3′
Sphingobacterium multivorum GIN723	Forward	5′-ATATATGGATCCGATGGCAGATAATGTATATGACGCAATTG-3′
	Backward	5′-ATATATAAGCTTTTATAGGTTTCCTTTTTTCAGCTCACTG-3′

Table S2. Relative activity and specificities of glycosidic linkage and glycone

Substrate	Relative activity (%)
p-Nitrophenyl α-D-glucopyranoside	103
p-Nitrophenyl β-D-glucopyranoside	100
p-Nitrophenyl α-D-galactopyranoside	201
p-Nitrophenyl β-D-galactopyranoside	400

Table S3. Substrate structure

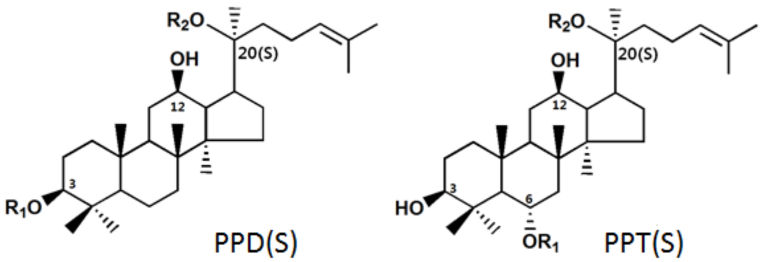

**Ginsenosides**	**R_1**	**R_2**
Ginsenoside- Rb1	-glu(2→1)glc	-glc(6→ 1)glc
Ginsenoside- Rb2	-glu(2→1)glc	-glc(6→ 1)arap
Ginsenoside- Rb3	-glu(2→1)glc	-glc(6→ 1)xyl
Ginsenoside- Rc	-glu(2→1)glc	-glc(6→ 1)araf
Ginsenoside- Rd	-glu(2→1)glc	-glc
20(S) Ginsenoside- Rg3	-glu(2→1)glc	-H
Ginsenoside- F2	-glc	-glc
Ginsenoside- Rh2	-glc	-H
Compound K	-H	-glc
Natoginsenoside Fe	-glu	-glc(6→ 1)araf
Compound Mc	-H	-glc(6→1)araf
Compound Y	-H	-glc(6→ 1)arap
Ginsenoside- Re	-glc(2→1)rha	-glc
Ginsenoside-F1	-H	-glc
Ginsenoside-Rg2	-glc(2→1)rha	-H
Gp-IX	-glu	-glc(6→ 1)xyl
**Other**	**Description**	
Icariin	4-O-methyl-8-γ,γ-dimethylallylkaempferol-3-rhamnoside-7-glucoside	
Icariside	3,5,8-Trihydroxy-2-(4-hydroxyphenyl)-4-oxo-4H-chromen-7-yl β-D-glucopyranoside	
Camelliaside A	Kaempferol3-O-(2-O-galactopyranosyl-6-O-rhamnopyranosyl)glucopyranoside	
Camelliaside B	Kampferol-3-O-(2-O-xylopyranosyl-6-O-rhamnopyranosyl)glucopyranoside	
